# Cardiac evaluation of paediatric athletes: A clinical consensus statement of the European Association of Preventive Cardiology (EAPC) of the ESC and the Association for European Paediatric and Congenital Cardiology (AEPC)

**DOI:** 10.1093/eurheartj/ehag188

**Published:** 2026-04-09

**Authors:** Guido E Pieles, Elena Cavarretta, Jessica J Orchard, Mark Abela, Elena Arbelo, Werner Budts, Silvia Castelletti, Guido Claessen, Domenico Corrado, Giovanni Di Salvo, Gherardo Finocchiaro, Peter Fritsch, Sabiha Gati, Stephan Gerling, M Cecilia Gonzalez-Corcia, Aneil Malhotra, Viviana Maestrini, Josef Niebauer, David Niederseer, Michael Papadakis, Renate Oberhoffer, Antonio Pelliccia, Nathan Riding, María Sanz-de la Garza, Georgia Sarquella-Brugada, Sanjay Sharma, Alan Graham Stuart, Monica Tiberi, Alessandro Zorzi, Flavio D’Ascenzi

**Affiliations:** Sports Cardiology and Screening Department, ASPETAR Qatar Orthopaedic and Sports Medicine Hospital, Sports City Street, Aspire Zone, PO Box 29222, Doha, Qatar; Faculty of Medical Sciences, University College London, Gower Street, WC1E 6BT, London, UK; Department of Medico-Surgical Sciences and Biotechnologies, Sapienza University of Rome, Latina, Italy; Advanced Cardiovascular Therapies Unit, Bambino Gesù Children's Hospital, IRCCS, Rome, Italy; Sydney School of Public Health, Faculty of Medicine and Health, The University of Sydney, Sydney, Australia; Department of Cardiology, Mater Dei Hospital, Msida, Malta; Faculty of Medicine, University of Malta, Msida, Malta; Arrhythmia Section, Cardiology Department, Hospital Clínic, Universitat de Barcelona, Barcelona, Spain; IDIBAPS, Institut d’Investigació August Pi i Sunyer (IDIBAPS), Barcelona, Spain; Centro de Investigación Biomédica en Red de Enfermedades Cardiovasculares (CIBERCV), Madrid, Spain; Institut D’Investigació August Pi I Sunyer (IDIBAPS) and European Reference Network for Rare, Low Prevalence and Complex Diseases of the Heart, ERN GUARD-Heart, Barcelona, Spain; Division of Cardiovascular Diseases, University Hospitals Leuven and Department of Cardiovascular Sciences, KU Leuven-University of Leuven, Leuven, Belgium; Cardiothoracic Department, University Hospital Santa Maria della Misericordia, Udine, Italy; Faculty of Medicine and Life Sciences/LCRC, UHasselt, Hasselt, Belgium; Hartcentrum Hasselt, Jessa Ziekenhuis, Hasselt, Belgium; Department of Cardiovascular Diseases, KU Leuven, Leuven, Belgium; Department of Cardiac, Thoracic and Vascular Sciences and Public Health, University of Padua Medical School, Padua, Italy; Department of Woman and Child Health, Paediatric Cardiology and Congenital Cardiology, University of Padua, Padua, Italy; Cardiology Research Centre, St George’s University of London, London, UK; Center for Pediatric Cardiology and Sportscardiology, Graz, Austria; Department of Cardiology, Royal Brompton & Harefield NHS Foundation Trust, London, UK; National Heart and Lung Institute, Imperial College, London, UK; Department of Pediatric Cardiology, University Children's Hospital Regensburg (KUNO), Hospital St.Hedwig of the Order of St. John, University of Regensburg, Regensburg, Germany; Pediatric Cardiology and Electrophysiology, Cliniques Universitaires Sainte Justine, University of Montreal, Montreal, Quebec, Canada; Institute of Sport, Manchester Metropolitan University, Manchester, UK; Department of Clinical, Internal, Anesthesiological and Cardiovascular Sciences, Sapienza University of Rome, Rome, Italy; Institute of Sport Medicine and Science, Italian National Olympic Committee, Rome, Italy; University Institute of Sports Medicine, Prevention and Rehabilitation and Research Institute of Molecular Sports Medicine and Rehabilitation, Paracelsus Medical University, Salzburg, Austria; Ludwig Boltzmann Institute for Digital Health and Prevention, Salzburg, Austria; Department of Cardiology, Hochgebirgsklinik Davos, Medicine Campus Davos, Davos, Switzerland; Department of Cardiology, Center for Translational and Experimental Cardiology (CTEC), University Hospital Zurich, University of Zurich, Zurich, Switzerland; Christine Kühne—Center of Allergy Research and Education (CK-CARE), Medicine Campus Davos, Davos, Switzerland; Cardiology Research Centre, St George’s University of London, London, UK; St George's University Hospitals NHS Foundation Trust, London, UK; Cleveland Clinic, London, UK; Chair of Preventive Paediatrics, Department of Sport and Health Sciences, Technical University of Munich, Munich, Germany; Institute of Sport Medicine and Science, Italian National Olympic Committee, Rome, Italy; Faculty of Medical Sciences, University College London, Gower Street, WC1E 6BT, London, UK; Population Health Sciences and Medical Schools, University of Bristol, Bristol, UK; Cardiovascular Institute, Hospital Clinic de Barcelona and Institut d'Investigacions Biomèdiques August Pi i Sunyer (IDIBAPS), Barcelona, Spain; Pediatric Arrhythmias, Inherited Cardiac Diseases and Sudden Death Unit, Hospital Sant Joan de Déu, Universitat de Barcelona, Barcelona, Spain; Cardiology Research Centre, St George’s University of London, London, UK; St George's University Hospitals NHS Foundation Trust, London, UK; Cleveland Clinic, London, UK; Bristol Congenital Heart Centre, The Bristol Heart Institute, University Hospitals Bristol NHS Foundation Trust, Bristol, UK; Department of Health, Sports Medicine Outpatient Clinic, Azienda Sanitaria Territoriale Pesaro-Urbino, Italy; Department of Cardiac, Thoracic and Vascular Sciences and Public Health, University of Padua Medical School, Padua, Italy; Department of Medical Biotechnologies, Sports Cardiology and Rehab Unit, University of Siena, Siena, Italy

**Keywords:** Sudden cardiac death, Screening, Paediatric athletes, Electrocardiogram

## Abstract

Paediatric athletes are not simply ‘mini adults’. Most existing recommendations for cardiac screening in paediatric athletes are primarily based on evidence in adults and are designed for adult athletes. Paediatric-specific recommendations are needed due to the specifics of cardiac physiology, maturation and growth, age-related disease expression, modified diagnostic pathways, training adaptations, and to address relevant ethical considerations. This clinical consensus document from the European Association of Preventive Cardiology (EAPC) of the ESC and the Association for European Paediatric and Congenital Cardiology (AEPC) introduces specific advice for paediatric athletes for the first time, based on expert consensus, and where available, data from paediatric athlete populations. Members of the writing group voted anonymously on key advice statements, with ≥80% agreement required for consensus. All advice in this document applies to paediatric athletes aged <16 years, including those under 12 years of age. This document advises that cardiac screening of paediatric athletes with personal and family medical history, physical examination and 12-lead resting electrocardiogram (ECG) should be performed and should start no later than the age of 12 years. Implementing a screening programme requires ensuring the availability of necessary healthcare resources. One transthoracic echocardiogram may be appropriate to identify high-risk structural cardiac diseases not identifiable on ECG, provided appropriate infrastructure for baseline diagnostic assessments is in place. This document also includes suggested definitions of normal, borderline and abnormal ECG findings in paediatric athletes. Detailed advice is provided for further evaluation if suspicious findings are identified on initial tests. This document highlights that further research is required to optimise screening strategies, accurately assess and quantify the risk of sudden cardiac death and provide evidence-based eligibility recommendations for paediatric athletes with cardiac disease. It is also noted that increased opportunities for paediatric sports cardiology training are required to provide adequate medical care for the paediatric athlete population.

## Graphical Abstract

**Graphical Abstract:**
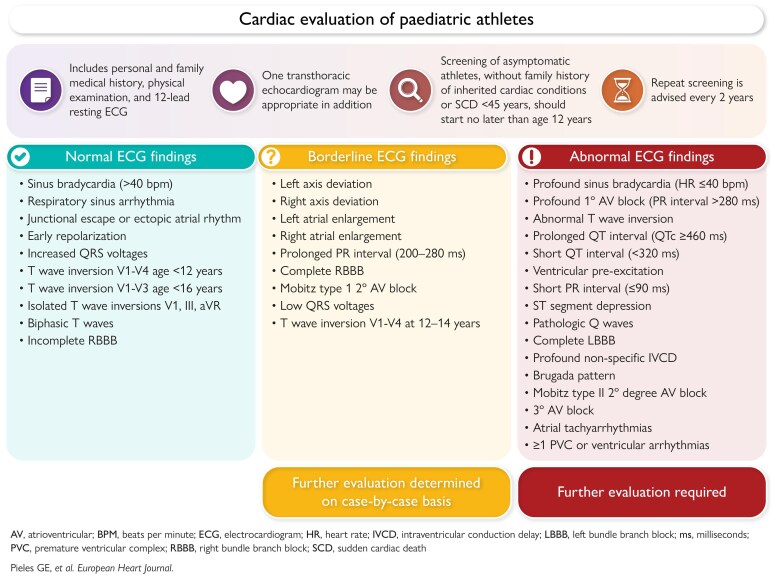
Advice for cardiac evaluation of paediatric athletes and suggested definitions of normal, borderline and abnormal electrocardiogram findings in paediatric athletes.

## Introduction: the rationale for cardiac screening advice specific to the paediatric athlete

Paediatric athletes are not simply ‘mini adults’. While paediatric athletes do display physiological cardiac remodelling in response to exercise,^1^ distinguishing between physiology and pathology may be more difficult due to maturational development and variable disease penetrance. However, most current cardiac screening guidelines and policies focus on adult athletes. A recent review of athlete screening guidelines found very few provided specific advice for paediatric athletes, and that further data are needed from studies specific to paediatric athletes.^2^ Further, the authors emphasise the importance of building additional paediatric cardiology expertise in the care of young athletes.^2^

A ‘paediatric athlete’ is usually defined as an individual aged 12–16 years participating in a team or individual sport with regular competition where there is a ‘high premium on excellence’ that involves regular, often intense, training.^2,3^ Defining athletic status in terms of age of starting competitive activity, training hours, intensity, and level of performance is useful when assessing the degree of exercise-induced cardiac remodelling. The reason athletes are targeted for cardiac screening is based on the notion that among those who have an underlying predisposition for a heart condition, exercise *per se* may act as a trigger for cardiac arrhythmia and the potential for sudden cardiac arrest or death (SCA/D).^4^ There is evidence that training and competition can be stimuli to trigger phenotypic cardiac disease, leading to a higher risk of SCA/D in young competitive athletes compared to non-athletes and children.^5^

Although the data supporting the implementation of cardiac screening in paediatric athletes are similar to that in adults, the optimal paediatric screening strategy is still debated.^2^ Investigating children can be challenging because maturation and development, particularly during puberty, must be taken into account when differentiating between physiological adaptation, growth and pathology. A recent study of 22 324 children aged 7–18 years, who underwent consecutive preparticipation screening, showed cardiac disorders associated with SCA/D were identified in 0.3%, with approximately one-third of diagnoses made at the first screening and two-thirds from subsequent examinations.^6^ Over 90% of the diseases were diagnosed in athletes aged 12 years or older.^6,7^

Specifically, the target population of this document is defined as asymptomatic athletes <16 years, including those younger than 12 years of age, without cardiovascular conditions, such as congenital heart diseases (CHD) or inherited cardiac conditions already identified. In these latter cases, the specific European Society of Cardiology (ESC) guidelines must be followed.^8–10^

### Consensus process and strength of advice

This document is a collaboration between the European Association of Preventive Cardiology (EAPC) of the ESC and the Association for European Paediatric and Congenital Cardiology (AEPC). It follows the principles of the EAPC and AEPC scientific document committees in terms of evaluating evidence and providing advice. Clinical consensus statements were based on the strength of evidence, and consensus was reached by anonymous voting by members of the writing group using web-based software (Qualtrics). At least 80% agreement was required for acceptance, although authors with dissenting opinions were able to have these views included in the manuscript.

The strength of advice is based on the following definitions in accordance with the ESC policy on consensus statements:

**Table:** 

Clinical advice, based on robust published evidence	****
Clinical advice, based on the uniform consensus of the writing group	***
May be appropriate, based on published evidence	**
May be appropriate, based on the consensus of the writing group	*
Area of uncertainty	?

When a statement was based on robust evidence, it was designated 4 stars. When a statement was predominantly based on expert opinion, it was assigned 3 stars when all the authors agreed (‘uniform consensus’). Statements were assigned 1 or 2 stars when >80%, but not all authors were in agreement. The distinction between 1 star and 2 stars was based on ‘some published evidence’ or ‘consensus opinion’, respectively. If consensus could not be reached, a statement was assigned a question mark, indicating an area of uncertainty.

*Table 1* sets out the key clinical consensus statements providing screening advice for paediatric athletes, indicating the strength of advice. Dissenting votes for statements that reached agreement are provided in Supplement 1. All advice in this document applies to paediatric athletes <16 years, including those younger than 12 years of age. While in many healthcare systems and jurisdictions the paediatric age is defined as below the age of 18 years, based on evidence of cardiac adaptation to exercise, but also historical concepts and consensus of other recommendation and guideline papers on athlete screening, for athletes over the age of 16 years, adult documents should be used.

**Table 1:** Advice on cardiac screening for paediatric athletes

		Strength of advice
**Screening advice & which tests to include**	Cardiac screening of athletes is advised with personal and family medical history, physical examination and 12-lead resting ECG.	****
**Echocardiogram considerations**	One transthoracic echocardiogram may be appropriate in addition to identify high-risk structural cardiac diseases not identifiable on ECG (e.g. congenital anatomic coronary artery anomalies and aortic disease).	***
	If an echocardiogram is performed, it is important that the appropriate infrastructure for baseline diagnostic assessments is in place, as well as specific paediatric expertise and resources for appropriate follow-up are available.	***
**Age to commence**	It is advised that screening of paediatric athletes should start no later than the age of 12 years, which is a common starting age for most competitive sports. It is important to ensure the availability of required healthcare resources in implementing a screening program.	**
**Frequency of screening**	In healthy athletes, it is suggested to repeat screening every 2 years until the age of 16 years. After this, advice for adult athletes should be followed.	***

ECG, electrocardiogram.

**Figure 1:**
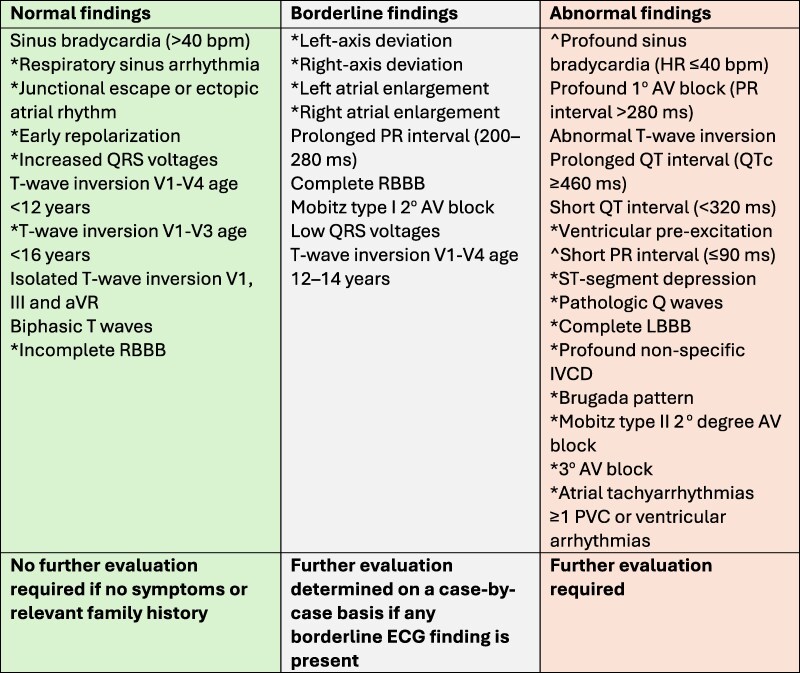
Paediatric athlete ECG findings. AV atrioventricular; IVCD, intraventricular conduction delay; LBBB, left bundle branch block; PVC, premature ventricular contraction; RBBB, right bundle branch block. Abnormal TWI: TWI in 2 or more leads avL, I, II, aVF; TWI in V5 or V6 even as single lead; TWI in V4 in athletes aged >14 years. *Indicates same category and definition as the International Criteria for Athlete ECG Interpretation (2017).^11^ ^ Indicates same category and definition as Ragazzoni (2023)^12^

*Figure 1* and the *Graphical Abstract* present a list of normal, borderline and abnormal ECG findings in paediatric athletes <16 years, including those younger than 12 years of age.

## Professionalism in adolescence and classification of athletes, exercise and sports

Establishing a habit of physical activity in childhood and adolescence has numerous health benefits, including improved cognitive function, bone and cardiometabolic health.^13^ The World Health Organisation recommends children aged 5–17 years undertake an average of 60 min/day of moderate to vigorous physical activity.^14^ While 81% of adolescents fail to meet the guidelines,^15^ 40–43% of children and adolescents worldwide participate in organised sport.^16^ This percentage is as high as 90% in Denmark, and around 60% in the USA.^16^

Where available, data show that 64% of participants in organised sports are under the age of 20 years, with 28% aged 10–14 years.^17^ Though conversion rates from youth to professional/elite sport are low (0.04% in football),^18^ there has been a continued drive towards early specialisation and professionalisation, with significant resources invested in identifying and developing future talented athletes.^19^ The increase in competitiveness, training loads, and competitive volumes without adequate time for recovery leads to concerns of an increased prevalence of injuries and health problems.^20^

The International Olympic Committee and the American Academy of Paediatrics have cautioned against early sports specialisation and the year-round focus on one sport.^20^ Nevertheless, in the United Kingdom, the football performance pathway starts at the age of 8, with those at 13 years of age undertaking 10–12 h of weekly training. In traditional early specialisation sports such as gymnastics and swimming, paediatric athletes regularly undertake 15 + hours of training per week.^21^ A study of elite college athletes showed that although early sport specialisation did confer increased risk of injury, such athletes were more likely to gain a scholarship, further driving early childhood talent identification and professionalisation.^22^

In addition to traditional sports, a new category of electronic sport (known as e-sports, esports, or exergames) is gaining momentum. Professional gamers often practice for many hours per day and participate in dedicated international competitions. Participants demonstrate increases in metabolic rate, energy expenditure and cardiopulmonary function, although the exercise physiology and cardiovascular function of e-athletes remains poorly studied.^23^ Emerging adverse events related to e-sports include internet gaming disorders, mental health and emotional impairments, long-lasting screen vision, parasympathetic inhibition, increased food intake, sleep deprivation, impairment in heart rate variability and potential cardiovascular risks.^24^ No specific requirements or a formal pre-participation screening for e-sport players have been proposed by scientific societies, and the research in this emerging area is still very limited. In a recently published proposal,^24^ the preparticipation screening should include not only cardiovascular assessment for the prevention of exercise-induced cardiac death, but also ophthalmologic, neurologic and psychological assessment and a correct training plan and an injury prevention strategy should be included. Paediatric e-sport players may be at a higher risk of exposure to inappropriate content, including violence, bullying, internet addiction, isolation, exposure to unhealthy lifestyle habits, and sleep deprivation, compared to adult e-sport players, a more comprehensive evaluation may be appropriate, and we wish to raise awareness on this emerging area.

## Physiological remodelling vs cardiomyopathies

Diagnosis of cardiomyopathies and arrhythmias can be very challenging due to differences in their phenotypic expression in childhood. Inherited cardiomyopathies often remain asymptomatic and subclinical in childhood, showing a rapid increase in phenotypic presentation in the second decade of life.^8^ However, many inherited or acquired paediatric arrhythmic diseases can be successfully diagnosed by screening, including electrocardiogram (ECG) and family history,^25^ especially if performed by professionals with paediatric cardiology expertise.

Physiological remodelling of the paediatric athlete’s heart resembles the physiological adaptation in adults, with harmonious enlargement of all four cardiac chambers, it is quantitatively less prominent than in adults. While pre-pubertal children generally exhibit less significant cardiac remodelling,^1,3,26^ factors such as maturation, growth and widely varying training volumes and intensities can hinder a clear differentiation between physiology and pathology. Conversely, adolescents in the peri-pubertal or post-pubertal stages may already show features of the athlete’s heart, especially if engaged in high-intensity sports activity with a pronounced aerobic component (e.g. running or swimming). While it is true that training modalities influence the type of cardiac remodelling, with more pronounced eccentric hypertrophy in endurance sports, recent data have questioned the validity of the classic Morganroth hypothesis in adult athletes.^27^ Therefore, while an understanding of this concept can aid in the cardiac assessment of an athlete, it is less useful to strictly categorise cardiac adaptation in athletes by sporting discipline, especially in paediatric athletes, where adaptation is present, but often less pronounced.^1,28^

Several consensus documents and reviews aid in differentiating between an athlete's heart and cardiomyopathies, but the distinction between these conditions remains challenging.^11,27,29^ A cardiomyopathy may be suggested by a positive family history, symptoms reported by the athlete, abnormalities on physical examination, or alterations in the ECG. In children, these changes can be subtle and knowledge of the nuances in cardiac adaptation to exercise in different age groups and the distinct presentations of early cardiomyopathies can enhance diagnostic accuracy. Absolute values of left ventricular (LV) or right ventricular (RV) thickness and size are rarely helpful in detecting pathology. Z-scores employing an algorithm that accounts for height and weight and normative population data can be discriminative,^30^ as can modern functional echocardiography tools such as 2D strain, for both RV^31,32^ and LV^31^ cardiomyopathies. Consequently, in a young athlete with a positive family or past medical history or ECG repolarisation abnormalities, even a borderline-enlarged left ventricle may be indicative of a developing pathology. This underscores the importance of careful and longitudinal evaluation and a high index of suspicion when screening paediatric athletes, while avoiding false positive results. When in doubt, consultation with experts in paediatric cardiomyopathies is crucial.^33^

## Causes of sudden cardiac arrest or death in paediatric athletes and the scope of cardiac screening

SCA/D in children is rare, but accounts for up to 10% of mortality after the first year of life.^34^ In the US general population, the incidence of sudden cardiac death is the lowest in children aged 1–10 years (0.42/100 000), with a slight increase in those aged 11–18 years (0.52/100 000), and further increases up to the age of 34 years (2.76/100 000).^7^ Therefore, adolescence, particularly the pubertal growth spurt, is an important phase of rapid change that can facilitate the transition from conceived to overt clinical phenotypes of genetic diseases such as cardiomyopathies and channelopathies.^35^ This is also an age group where the intensity of sport and training requirements increases significantly.

The reported incidence of SCA/D in young athletes aged 8–15 years is 0.7/100 000/year.^36^ These tragic events are rarer in individuals aged 8–11 years, with the incidence rising in those aged 12–15 years (1.2/100 000/year).^36^ Often, there are no warning signs, with only a minority experiencing symptoms prior to death.^35^ An estimated 1 in 300 children engaged in competitive sport has a cardiac condition at risk of SCA/D.^37^

Sudden arrhythmic death syndrome (SADS), myocardial diseases and coronary artery anomalies are the most common aetiologies of SCA/D in adolescents. Children who suffer sudden death are more likely than adults to have a structurally normal heart at the post-mortem examination.^23^ In 52% of cases of sudden death in children aged <18 years, the post-mortem examination was consistent with SADS with a structurally normal heart, suggesting a possible underlying primary arrhythmia syndrome. In contrast, SADS occurred in 42% of adult athletes aged 18–35 and in 26% of adults >35 years.^38^ Coronary artery anomalies were the second most common cause of sudden death (17% of cases) in a large cohort of young American athletes.^39^

Cardiac conditions associated with SCA/D are often identified through screening with a personal/family history assessment and resting 12-lead ECG. The yield of screening is lower in younger children (0.05%),^6^ increasing to 0.1–0.3% in early adolescence.^6,40^ Serial screening appears to improve the diagnostic yield.^35,41,42^ For example, in an 11-year study of 22 324 children aged 7–18 years who underwent serial cardiac screening, 69 cases (0.3%) of conditions associated with SCA/D were identified, but 91% of these were identified in children ≥12 years, and 64% were identified only on repeat evaluation.^6^ The rise in SCA/D during adolescence^43^ is an argument in favour of screening in peri-pubertal individuals. In addition, recent studies have shown that young individuals with cardiomyopathies (10–15 years of age) are at higher risk of SCA/D during exercise compared to adults with the disease.^35,44^

However, there are some caveats which must be taken into account when screening this age group. First, accurate interpretation requires specific expertise in paediatric athlete ECGs.^12^ For example, anterior T-wave inversion (TWI) beyond V2, which is currently regarded as an abnormal feature in most adult athletes, is a common peri-pubertal physiological phenomenon, especially before the pubertal growth spurt.^45–48^ Secondly, screening in adolescents has a lower diagnostic yield compared to older athletes, where the phenotypic expression of heritable cardiac disease may be clearer and more overt. Finally, screening in this age group leads to a greater number of ‘grey’ cases compared to older athletes. ‘Grey cases’, and all abnormal results (including false positives), require further evaluation, which has implications in terms of cost, time and potential anxiety for the athlete and their family. Strategies to reduce the burden of false positive results include: accurate interpretation of ECGs in accordance with paediatric-specific athlete criteria, providing a clear pathway for those requiring further evaluation, and ensuring all follow-up tests are completed and communicated in a timely manner.^49^ Conversely, even a best practice programme will miss some cases, as not all conditions are visible on an ECG, or may vary over time.^50^ It is important to inform athletes (and their parents) of the limitations of screening and to report any future symptoms.

Marked heterogeneity exists across different countries in paediatric cardiac screening practices and protocols, as there are insufficient data to support a particular strategy.^25^ Some countries conduct broad, general screening examinations of all children, commencing at the age of 6 years, and repeated at age 12.^51^ These examinations may or may not include an ECG. Italy introduced a programme of ECG screening of newborns in the 4th week of life for long QT syndrome, which causes approximately 10% of sudden infant deaths.^52^ Several countries have implemented systematic screening of community-level athletes, often commencing in early adolescence. In Italy, this mandatory screening includes an ECG and simplified stress ECG, with commencement age depending on the sport (usually between 8 and 12 years).^6^ In contrast, many countries have no systematic screening programmes for paediatric community athletes or the general school-aged population, meaning that the only screening programmes are conducted by sporting organisations and generally limited to elite athletes.^25^

## Screening modalities, age ranges and screening frequency in paediatric athletes

**Figure 2:**
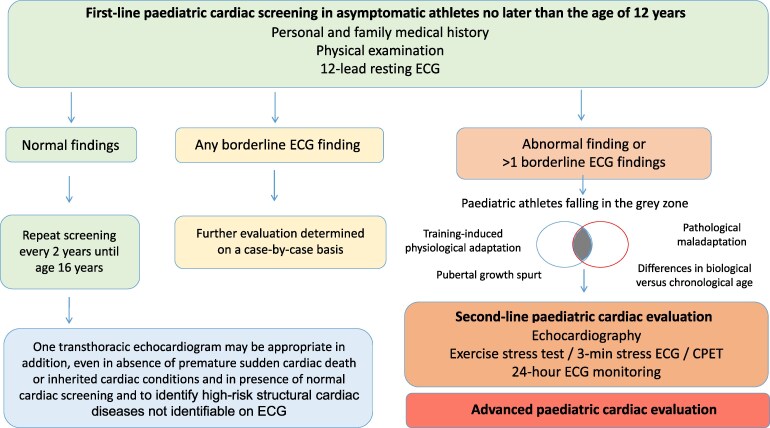
Screening and secondary investigations. CPET, cardiopulmonary exercise test; ECG, electrocardiogram

This document advises that cardiac screening of paediatric athletes with personal and family medical history, physical examination and 12-lead resting ECG should be performed and should start no later than the age of 12 years (*Figure 2*). All advice in this document applies to paediatric athletes <16 years, including those younger than 12 years of age. The age of 12 years is a common starting age for most competitive sports, the age at which inherited cardiomyopathies may manifest structurally, and when there is a discernible increase in SCA/D in population studies.^25,36^ It is noted that some countries conduct national screening programmes which start earlier than 12 years of age or between the ages of 14–16 years.^25^ Countries with these programmes can continue to follow national guidance. Implementing a screening programme requires ensuring the availability of necessary healthcare resources.

One transthoracic echocardiogram may be appropriate to identify high-risk structural cardiac diseases not identifiable on ECG (e.g. congenital anatomic coronary artery anomalies, valve disease and aortic disease). If an echocardiogram is performed, it is important that the appropriate infrastructure for baseline diagnostic assessments is in place, as well as availability of specific paediatric expertise and resources for appropriate follow-up.

In healthy athletes, repeat screening is advised every 2 years until the age of 16 years. After this, advice for adult athletes should be followed.^10^

If definitive or suspicious pathological findings are identified on the preliminary screening tests, an echocardiogram and stress ECG are advised as first-line examinations, as clinically indicated.

*Table 2* sets out the indications for performing an echocardiogram in paediatric athletes.^53^ Structural and functional echocardiograms should be performed in accordance with international paediatric recommendations.^54^ Performing and interpreting echocardiography in paediatric athletes requires expertise in congenital and paediatric conditions.

**Table 2:** Reasons for transthoracic echocardiography in paediatric athletes

Echocardiogram reason	Details
General advice/requirements: Specific expertise in paediatric echocardiography is required Echocardiograms should be performed in accordance with paediatric and athlete recommendations Z-scores should be used for interpretation	
Abnormal ECG findings	Echocardiogram indicated, see *Figure 2*
Borderline ECG findings	Advised on a case-by-case basis
Personal history of CV symptoms at rest or during effort	Unexplained syncope Unexplained chest pain on exertion that is unlikely to be musculoskeletal Palpitations, unexplained dyspnoea at effort
Abnormal physical examination (murmurs or stigmata of inherited cardiac disease)	Diastolic murmur Systolic murmur ≥2/6 Murmur with radiation or holosystolic or unchanged with position Murmur that is accentuated with the Valsalva manoeuvre Stage 1 hypertension at rest Differential blood pressure increase Reduced/differential pulses Suspicion of connective tissue disorder or syndromic disease
Family history of cardiomyopathies or sudden cardiac death <40 years old	First or second degree relative with SCA/D aged <40 years (excluding myocardial infarction) First or second-degree relative diagnosed with cardiomyopathy or a condition associated with SCA/D Non-cardiac congenital malformations First-degree history of congenital heart disease (familial recurrence 5–15%)

CV, cardiovascular; ECG, electrocardiogram; SCA/D, sudden cardiac arrest/death.

## Family history and examination—structured assessment

A structured approach to clinical cardiovascular assessment of the paediatric athlete should include an exercise and training history, personal and family medical history and physical examination. Parents and guardians should be fully informed of the screening process, and their involvement provides the opportunity to identify major issues, build rapport and provide education. However, screening with personal/family history and physical examination alone has been reported to have a sensitivity of around 20% and specificity of 68%, both of which are substantially lower than when an ECG is included.^37,55^ The current International Criteria have a sensitivity of 97–99% and specificity of 86% in older adolescent soccer players aged 16.4 ± 1.2 years.^56^ In athletes aged 12–14 years, the current International Criteria have a lower sensitivity (57%), but a higher specificity (98%).^57^

The medical history should enquire about exercise and non-exercise related cardiovascular symptoms such as chest pain, disproportionate dyspnoea on exercise, palpitations or syncope. The history should include current medications, including supplements, and details of major illnesses requiring hospital admission or surgery. Common conditions, such as asthma, should be noted, as they may overlap with symptoms of cardiac disease. Details of the athlete’s exercise history and current exercise participation should include type, frequency, intensity and duration of exercise sessions. Sleep quality and duration, as well as the amount of screen time, should be assessed.

A family history should be taken, concentrating on the presence of early onset cardiovascular disease in a first or second degree relative, including inherited cardiomyopathies and arrhythmia syndromes, ischaemic heart disease, aortic dissection, connective tissue disorders, diagnosis of a condition associated with SCA/D, exercise-related arrhythmias and syncope and SCA/D in a relative aged <40 years. Such details are important, but may not always be available. Thus, lay terms such as ‘heart attack’ may be applied to many conditions ranging from myocardial infarction to sudden arrhythmic death. The implantation of a pacemaker may actually refer to an implantable defibrillator. Similarly, unexplained car accidents or deaths while swimming can be due to an arrhythmic cause.

If the athlete has a first-degree relative affected by an inherited cardiac condition with a pathogenic/likely pathogenic variant identified, specific guidelines for pre-test genetic counselling should be followed.^8,33^

A cardiovascular examination should be conducted, including measuring blood pressure, palpation of dorsal pedis pulses to exclude aortic coarctation and a general examination for signs of a syndromal anomaly that might have cardiovascular implications, e.g. Noonan or Marfan’s syndrome (*Table 2*).

## Resting ECG: normal, borderline and abnormal values, physiological and age-related changes in paediatric athletes

ECGs in paediatric athletes demonstrate both physiological adaptation of the heart to regular exercise and growth and maturation.^1,58^ This is reflected in parameters such as lower resting heart rate, longer PR interval and QRS duration, and changes in repolarisation.^58–61^ Several common ECG findings in adult athletes are also present in paediatric athletes, such as sinus bradycardia, incomplete right bundle branch block (RBBB), anterior TWI, first-degree heart block and early repolarisation.^58,62^

Normative values and expression of ECG variables by z-scoring have been proposed for children and may help in high-volume screening.^63^ A detailed understanding of these changes is critical for distinguishing between benign training-induced remodelling and potentially life-threatening cardiac conditions. However, while the International Criteria for Athlete ECG Interpretation have been validated in adults, they have a lower sensitivity in paediatric athletes.^64^ A revision of the International Criteria was recently proposed and adapted for paediatric athletes, although it requires validation and refinement.^12^

A list of suggested ECG recommendations for paediatric athletes is presented in *Figure 1*.

*Table 3* provides proposed definitions of normal ECG findings in paediatric athletes. In the absence of symptoms or relevant family history, the following ECG parameters are regarded as *normal* in paediatric athletes and do not require any further evaluation.

**Table 3:** Normal ECG findings in asymptomatic paediatric athletes in the absence of a family history

ECG feature	Proposed definition of normal value/finding for paediatric athletes
Sinus bradycardia	*Heart rate >40 bpm*
Respiratory sinus arrhythmia^a^	*Sinus arrhythmia* A normal variant in the absence of symptoms such as dizziness, syncope, or fatigue
Junctional escape or ectopic atrial rhythm^a^	*QRS rate is faster than the resting P wave or sinus rate and typically less than 100 bpm with a narrow QRS complex unless the baseline QRS is conducted with aberrancy*. A normal variant in the absence of symptoms such as dizziness, syncope, or fatigue. Sinus rhythm should return with a brief episode of brisk exercise.
Early repolarisation^a^	*J-point elevation, ST-segment elevation, J waves or terminal QRS slurring in the inferior and/or anterior and lateral leads in all/any leads*
Increased QRS voltages^a^	*Isolated QRS voltage criteria for left (SV1 + RV5 or RV6 > 3.5 mV) or right ventricular hypertrophy (RV1 + SV5 or SV6 > 0.8 mV)* These are primarily due to a low transthoracic impedance and, in the context of an otherwise normal ECG, should be regarded as a normal finding.
T-wave inversion	*Juvenile patterns:* V1-V3 in athletes aged <16 years^a,b^ V1-V4 in athletes aged <12 years *Isolated V1, III and aVR* *Biphasic T waves*
Incomplete RBBB^a^	*rSR’ pattern in V1 and qRS pattern in V6 with QRS duration <120 ms*

ECG, electrocardiogram; RBBB, right bundle branch block.

^a^Indicates same definition as the International Criteria for Athlete ECG Interpretation (2017).^11^

^b^Indicates same category and definition as Ragazzoni (2023).^12^

*Table 4* sets out *borderline* ECG findings in paediatric athletes. Further evaluation is determined on a case-by-case basis if one or more borderline findings are present. This recognises that not all borderline findings definitely require further evaluation and allows individual discretion and judgement, reflecting current clinical practice.

**Table 4:** Borderline ECG findings in asymptomatic paediatric athletes in the absence of a family history

ECG feature	Proposed definition for paediatric athletes
Left-axis deviation^a^	*−9° to −90°*
Right-axis deviation^b^	*>120°*
Left atrial enlargement^b^	*Prolonged P-wave duration of >120 ms in I or II, with a negative portion of the P wave ≥1 mm in depth and ≥40 ms in duration in V1*
Right atrial enlargement^b^	*P wave ≥2.5 mm in II, III or aVF*
Prolonged PR interval	*PR interval 200–280 ms*
Complete RBBB^b^	*rSR’ pattern in V1 and S wave wider than R wave in V6 with QRS duration ≥120 ms*
Mobitz type I (Wenckebach) 2^°^ AV block	*PR interval progressively lengthens until there is a non-conducted P wave with no QRS complex; the first PR interval after the dropped beat is shorter than the last conducted PR interval.*
Low QRS voltages	*QRS amplitude from peak to nadir <0.5 mV in all limb leads*
T-wave inversion	*T-wave inversion V1-V4 age 12–14 years*

AV, atrioventricular; ECG, electrocardiogram; RBBB, right bundle branch block.

^a^Indicates same category and definition as Ragazzoni (2023).^12^

^b^Indicates same definition as the International Criteria for Athlete ECG Interpretation (2017).^13^

The ECG parameters in *Table 5* are regarded as *abnormal* in paediatric athletes and further evaluation is advised (*Table 6*).

**Table 5:** Abnormal ECG findings in paediatric athletes

ECG abnormality	Proposed definition for paediatric athletes
Profound sinus bradycardia^a^	*HR < 40 bpm*
Profound 1^°^ AV block	*PR interval >280 ms*
Abnormal T-wave inversion	*Age <16 years:* *TWI in 2 or more leads: V4,* ^b^ *V5, V6, I, II, aVL, aVF; or TWI in V5 or V6 as single leads* *Age <12 years:* *TWI in 2 more leads: V5, V6, I, II, aVL, aVF; or* *TWI in V5 or V6 as single leads*
Prolonged QT interval	*QTc ≥460 ms in all genders* *Use Fridericia correction if HR <60 bpm or >80 bpm*
Short QT interval	*QTc <320 ms*
Ventricular pre-excitation^a^	*PR interval ≤90 ms with a delta wave and wide QRS (≥90 ms)*
ST-segment depression^c^	*≥0.5 mm in depth in ≥2 contiguous leads*
Pathologic Q waves^c^	*Q/R ratio ≥0.25 or ≥40 ms in duration in ≥2 leads (excluding III and aVR)*
Complete LBBB^c^	*QRS ≥120 ms, predominantly negative QRS complex in lead V1 (QS or rS) and upright notched or slurred R wave in I and V6*
Brugada pattern^a^	*Coved-type ST-segment elevation ≥2 mm in ≥1 right precordial lead*
Mobitz type II 2^°^ AV block^a,c^	*Intermittently non-conducted P waves with a fixed PR interval*
3^°^ AV block^a,c^	*Complete heart block*
Atrial tachyarrhythmias^a,c^	*Supraventricular tachycardia, atrial fibrillation, atrial flutter*
Ventricular arrhythmias	*≥1 PVC, couplets, triplets and non-sustained ventricular tachycardia*

AV, atrioventricular; HR, heart rate; LBBB, left bundle branch block; PVC, premature ventricular contraction; TWI, T-wave inversion.

^a^Indicates same category and definition as Ragazzoni (2023).^12^

^b^T-wave inversion V1-V4 in athletes aged 12–14 years is categorised as a borderline finding.

^c^Indicates same definition as the International Criteria for Athlete ECG Interpretation (2017).^11^

**Table 6:** ECG abnormalities and suggested further evaluation

ECG abnormality	Possible diagnosis	Suggested tests	Further considerations
Profound sinus bradycardia (HR ≤40 bpm)	Cardiac dysfunction, sinus node dysfunction, conduction disease, channelopathies	Echocardiogram, exercise stress ECG, 24 h ECG	CMR if echo abnormal
Profound 1^°^ AV block (PR interval >280 ms)	Conduction disease	Echocardiogram, stress ECG, 24 h ECG	Rarer disease (immune, infection)
Abnormal T-wave inversion	Cardiomyopathy, myocarditis, abnormal origin of CA	Echocardiogram, stress ECG, 24 h ECG	CMR if suspicion or abnormal findings; genetic referral and testing if signs of disease or family history
Prolonged QT interval (QTc ≥460 ms)	Long QT syndrome, electrolyte abnormalities, and medication	Echocardiogram to rule out structural abnormalities, stress ECG, 24 h ECG, blood electrolytes	ECG in 1st degree family members, genetic referral and testing if high suspicion or signs of long QT syndrome
Short QT interval (<320 ms)	Short QT syndrome, electrolyte abnormalities, and medication	Echocardiogram to rule out structural abnormalities, stress ECG, 24 h ECG, blood electrolytes	1st degree family ECG, genetic referral and testing if high suspicion or signs of short QT syndrome
Ventricular pre-excitation	WPW syndrome	Echocardiogram, stress ECG, 24 h ECG	Referral to an electrophysiologist
ST-segment depression	Cardiomyopathy, myocarditis, abnormal origin of CA (ischaemia)	Echocardiogram, stress ECG	CMR if suspicion or abnormal findings
Pathologic Q waves	Cardiomyopathy, myocarditis, CAD	Echocardiogram, stress ECG	CMR if suspicion or abnormal findings
Complete LBBB	Cardiomyopathies, Noonan syndrome, coronary artery anomalies, complex CHD pre- and post-surgical correction	Echocardiogram, stress ECG, 24 h ECG (for arrhythmia detection)	CMR if suspicion or abnormal findings
Profound non-specific IVCD	Cardiomyopathies, myocarditis, conduction disease	Echocardiogram, stress ECG, 24 h ECG	
Brugada pattern	Brugada syndrome, medication	Echocardiogram, stress ECG, 12-lead 24 h ECG	Genetic referral and testing if high suspicion; 1st degree family ECG if high suspicion; referral to electrophysiologist.
Mobitz type II 2° AV block	Congenital HB, immune disease, infection	Echocardiogram, stress ECG, 24 h ECG	Referral to an electrophysiologist, additional tests (e.g. autoimmune blocks) for specific cases
3^°^ AV block	Congenital HB, immune disease, infection	Echocardiogram, stress ECG, 24 h ECG	Referral to an electrophysiologist.
Atrial tachyarrhythmias	Acquired or congenital electrical disease	Echocardiogram, stress ECG, 24 h ECG	Referral to an electrophysiologist.
≥1 PVC or ventricular arrhythmias	Cardiomyopathy, myocarditis, electrical disease	Echocardiogram, stress ECG, 24 h ECG	CMR if suspicion or abnormal findings, uncommon morphology or complex VAs; referral to an electrophysiologist.

ARVC, arrhythmogenic right ventricular cardiomyopathy; AV, atrioventricular; CA, coronary artery; CAD, coronary artery disease; CHD, congenital heart disease; CMR, cardiac magnetic resonance; ECG, electrocardiogram; HB, heart block; HR, heart rate; IVCD, intraventricular conduction delay; PVC, premature ventricular contraction; VA, ventricular arrhythmia; WPW, Wolff-Parkinson-White.

### Profound sinus bradycardia

The cut-off for marked bradycardia differs between paediatric and adult athletes. For paediatric athletes, a threshold of 40 bpm is proposed.^12^ Therefore, heart rates >40 bpm are regarded as normal in paediatric athletes, if in sinus rhythm. Beyond bradycardia, respiratory sinus arrhythmia, junctional escape rhythm, and isorhythmic dissociation may be found in paediatric athletes due to increased vagal tone. These are normal variants in the absence of symptoms such as dizziness, syncope, or fatigue. In borderline cases, mild aerobic activity at the bedside or formal exercise testing can be useful to demonstrate normal sinus node activity with restoration of sinus rhythm and a normal chronotropic competence.

### Early repolarisation

Early repolarisation is common in paediatric athletes. Although it is more prevalent in athletes (and particularly in those engaged in endurance sports), it is also found in sedentary children due to age-related enhanced vagal tone. All patterns of early repolarisation are regarded as normal, and no further investigations are needed in the absence of symptoms or family history.

### T-wave inversion

The interpretation of repolarisation can be particularly challenging in children: anterior TWI is commonly observed and regarded as a normal age-related pattern due to right ventricular dominance.^65^ However, anterior/chest lead TWI, particularly when extending to V4, may also indicate an early cardiomyopathy.^47^ The prevalence of anterior TWI starts decreasing as individuals progress from childhood to adolescence and young adulthood.^46,48,66^ Anterior TWI extending beyond lead V2 is rare (0.1%) in white athletes aged ≥16 years or younger athletes who have completed puberty; in these cases, further investigations might be needed to exclude the presence of early cardiomyopathy.^46,47^ Recent studies in children have shown that reversal to positive anterior T waves is correlated with chronological and biological age,^1^ and also with height and weight.^59^ A study in 2151 athletes showed variations in ECG findings depended on age and training load, suggesting the use of reference values to discriminate between true and false positive findings.^2,61^

In children with incomplete pubertal development, anterior TWI should not prompt further evaluation in the absence of symptoms, other ECG abnormalities or positive family history.^11^ Therefore, isolated TWI in leads V1-V4 in paediatric athletes until the age of 12 years is regarded as a normal finding, but is classified as a borderline finding in those aged 12–14 years. Pubertal stage has a major influence on TWI,^28^ however, routine assessment of pubertal stage during the cardiac screening encounter is not advised as it is impractical and potentially extremely uncomfortable for the athlete. Ethnicity/genetic background may impact the prevalence of patterns of TWI in the healthy paediatric athlete, but current data do not support a classification based on ethnicity/genetic background.^67^ T-wave morphology remains an important criterion to differentiate between physiology and pathology, e.g. deep TWI with isoelectric ST segments that do not follow normal R-wave progression (reduction of negative T-wave amplitude from right anterior to lateral leads) are much less likely benign and need to be investigated.

**Figure 3:**
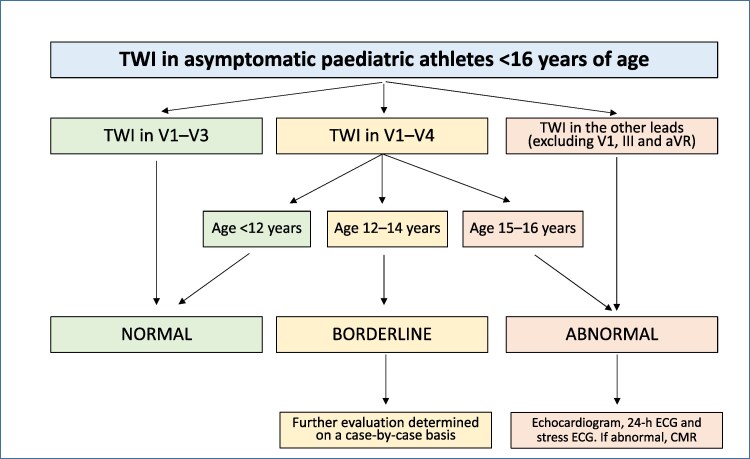
Normal, borderline and abnormal T wave inversion in paediatric athletes. CMR, cardiac magnetic resonance; ECG, electrocardiogram; TWI, T-wave inversion. Chronological age is often different from biological age, and this will impact repolarisation (see McClean *et al*^28^); biphasic T waves are not an abnormal finding; isolated TWI in V1, III and aVR is always regarded as normal.

*Figure 3* sets out the definitions of normal, borderline and abnormal TWI in paediatric athletes. TWI in leads III and aVR is always regarded as normal. Biphasic T waves (in isolation) are also regarded as normal.

### Left and right-axis deviation

Left-axis deviation (−9° to −90°)^12^ and right-axis deviation (>120°)^11^ may indicate underlying structural heart disease, such as hypertrophic cardiomyopathy (HCM), valve abnormalities, or conduction disease.^68^ However, right-axis deviation is often a normal finding in pre and peripubertal children and should be treated as a borderline change, warranting further investigation if other signs of potential disease or clinical suspicion of disease are present.

### PR interval

While PR interval increases with age, the presence of first-degree atrioventricular (AV) block (i.e. a PR interval >200 ms), although still physiological in many cases, is not a very common finding in peri-pubertal athletes (0.4%).^61^ Therefore, a PR interval of 200–280 ms is classified as a borderline finding.

However, a prolonged PR interval of more than 280 ms in the paediatric athlete (vs ≥400 ms in adult athletes)^12^ should be further investigated as it may be due to sinus node dysfunction and may represent a sign of acquired or congenital conduction system dysfunction.^12^

For cases of advanced AV block (higher than second-degree Mobitz type I), additional tests such as autoimmune blocks may be appropriate for specific cases.^69,70^

A short PR interval ≤90 ms is regarded as abnormal in paediatric athletes.^12^

### Complete right bundle branch block

Complete RBBB has been categorised as a borderline finding, as it can be present in healthy paediatric athletes and is not a risk factor for SCA/D. However, it can also be associated with CHD, resulting in intracardiac shunts such as Ebstein’s anomaly or atrial septal defects.^12^ Therefore, further evaluation with echocardiography is suggested when a complete RBBB is found during the first clinical evaluation to exclude significant congenital heart disease.

This document uses the current definition of complete RBBB with a QRS duration of ≥120 ms, but it has been reported that pathology is more frequently identified in individuals with complete RBBB and QRS duration ≥130 ms,^71^ so the QRS duration may be relevant in determining further evaluation.

### Left and right atrial enlargement

Left atrial enlargement can be a sign of increased LV pressures and volume, mitral and aortic valvar pathology. Left atrial enlargement has been classified as a borderline finding as it is non-specific and non-discriminatory in children, and normal cut-off values are not well defined.

Right atrial enlargement can be a sign of pulmonary arterial hypertension, increased RV pressures and volume, tricuspid and pulmonary valvular pathology. Right atrial enlargement has been classified as a borderline finding when in isolation, as it has a low positive predictive value in children^72^ and the criteria are not very well defined. Furthermore, it is not an isolated risk factor for SCA/D.

### Low QRS voltages

Low QRS voltages, defined as QRS amplitude from peak to nadir <0.5 mV in all limb leads^73^ have been categorised as a borderline finding. Low QRS voltages are rare in paediatric athletes (0.2%)^74^ and are not related to age, sex, type or the intensity of the sporting discipline, but equally are they not necessarily associated with cardiac disease. The presence of low QRS voltage may prompt further investigations to exclude the presence of structural heart disease, especially if coupled with fragmented QRS in ≥2 contiguous leads excluding V1–V2,^74–77^ or associated with signs or symptoms of cardiac disease.

### Mobitz type I (Wenckebach) second-degree atrioventricular block

In paediatric athletes, second-degree heart block is very rare and has not been described as a feature of training adaptation, contrary to adult athletes, where it is regarded as a normal variant.^11^ Therefore, it has been categorised as a borderline finding, and investigations may be appropriate if suspicion of a primary or secondary conduction anomaly exists.

### Prolonged QT interval

The QTc may be relatively prolonged in paediatric athletes; however, a value ≥460 ms is uncommon even in high-level junior athletes. Usually, the QT interval is corrected with the Bazett formula. However, in cases where the heart rate is <60 bpm or >80 bpm, the QT interval should be corrected using both the Bazett and Fridericia correction formulae. The Fridericia formula has been shown to perform better in terms of removing the effect of heart rate, especially at lower and higher heart rates (e.g. ≥80 bpm), in athletes and young people.^78,79^ It is important to note that the Bazett and Fridericia corrections are not interchangeable.^80^ At present, there are insufficient data available for sex-specific cut-off values in paediatric athletes.

### Short QT interval

A short QT interval, defined as a QTc <320 ms,^81^ may indicate a genetic condition that could predispose an individual to ventricular arrhythmias and SCA/D. Paediatric athletes with a short QT interval should be investigated.

### Pre-excitation (Wolff-Parkinson-White syndrome)

In adolescent athletes with pre-excitation, atrial fibrillation with a rapidly conducting pathway, in some cases, can cause a chaotic ventricular rhythm, leading to collapse and SCA/D. Differentiating benign from high-risk pathways is critical. Exercise testing can be helpful, but it has a suboptimal negative predictive value in adults.^82^ Referral to an electrophysiologist is advised in all cases and an electrophysiological study may be indicated.^29^

### ST-segment depression

ST-segment depression often signals underlying cardiomyopathy or ischaemic heart disease, uncommon in normal athletic adaptations. However, it can also be a sentinel of abnormal origins of the coronary arteries and resting echocardiography should be performed. If inconclusive, cross-sectional imaging is required to establish coronary artery origin. Exercise stress echocardiography may be appropriate to assess for wall motion abnormalities and exercise-associated dysfunction.

### Atrial tachyarrhythmias

Atrial tachyarrhythmias including atrial fibrillation, atrial flutter, ectopic atrial tachycardia, and AV (nodal) re-entry tachycardia are infrequent in paediatric athletes with structurally normal hearts, necessitating individualised management if present.^29^

### Ventricular arrhythmias

Ventricular arrhythmias are uncommon in paediatric athletes and are strongly associated with SCA/D. Cases warrant thorough investigation to rule out conditions like channelopathies, cardiomyopathies, myocarditis or structural congenital heart disease such as coronary artery abnormalities.

Premature ventricular contractions (PVCs) are often benign, especially when asymptomatic and monomorphic. However, PVCs that are symptomatic, frequent, or polymorphic can indicate underlying heart disease.^29^  *Table 7* sets out a classification of characteristics of low-risk and high-risk PVCs in junior athletes. In the paediatric athlete, any number of PVCs in the resting ECG should trigger further investigations with the aim to define the aetiology of the PVC^12^ (without over-investigating benign PVCs). Benign RV outflow tract tachycardia with PVC with LBBB morphology is common in paediatric athletes, but investigations for an underlying right-sided cardiomyopathy may be required, in particular if other disease criteria are present.^83,84^ Conversely, PVCs with an uncommon/high-risk morphology should be thoroughly investigated.^83,84^ An echocardiogram may also be appropriate if a PVC-induced cardiomyopathy is suspected.

**Table 7:** Classification of premature ventricular contractions in junior athletes according to their qualitative characteristics

	Common/low-risk	Uncommon/high-risk
**PVC characteristics**		
Morphology	LBBB/inferior axis RBBB and narrow QRS (<130 ms)	LBBB with intermediate axis or superior axis RBBB and wide QRS (>130 ms)
Response to exercise	Decrease/suppression	Persistence/increase
Complexity	Isolated, monomorphic	Repetitive, polymorphic
Short coupling interval (R on T)	No	Yes
Reproducibility at ET and/or serial ambulatory ECG monitoring	No	Yes
**Clinical findings**		
Exercise-related syncope/pre-syncope	No	Yes
Family history of premature SCA/D^a^ or cardiomyopathy	No	Yes
ECG abnormalities	No	Yes
Imaging abnormalities	No	Yes

ECG, electrocardiogram; ET, exercise testing; LBBB, left bundle branch block; PVC, premature ventricular contraction; RBBB, right bundle branch block; SCA/D, sudden cardiac arrest/death.

Adapted from Ragazzoni *et al* (2023).^12^

^a^Premature SCA/D is defined as that occurring before age 40 in males and before age 50 in females.

## Congenital arrhythmias and channelopathies, acquired arrhythmias.

### Channelopathies

Long QT syndrome has a prevalence of about 1 in 2000 in the general population.^52^ Typically, significant QTc prolongation is observed on the resting ECG.^6,41^ However, LQTS may present with a normal basal ECG, with QTc prolongation becoming apparent only during post-exercise recovery. In confirmed LQTS, vigorous exercise might be well tolerated if expert monitoring and treatment are provided.^85^ According to the 2020 ESC Guidelines on sports cardiology and exercise in patients with cardiovascular disease, participation in high-intensity recreational and competitive sports is not recommended for adult patients with QTc >500 ms or genetically confirmed LQTS with QTc ≥470 ms (males) or 480 ms (females).^10^ The latest American Heart Association/American College of Cardiology scientific statement does not preclude competitive sports in genotype-positive/phenotype-negative athletes and phenotype-positive athletes, following individualised risk assessment and decision making as well as medical preventive therapy.^86^

### Brugada syndrome

A spontaneous Brugada ECG pattern is rarely seen in children.^87^ However, an incomplete RBBB with slight ST-segment elevation may be the only sign of the underlying condition. Patients with Brugada syndrome^88^ without documented arrhythmias generally have no contraindication to sports activities, except in conditions of excessive heat, where adequate hydration and body temperature management strategies are paramount. A high lead 12-lead ambulatory ECG may also help rule out an intermittent Type 1 Brugada pattern.

### Catecholaminergic polymorphic ventricular tachycardia

Catecholaminergic polymorphic ventricular tachycardia is a possible diagnosis when ventricular ectopy is present, although the resting ECG and physical examination are usually normal. In cases where a channelopathy is suspected, clinical examination is often unremarkable, and the resting ECG may not reveal diagnostic information. Exercise testing is advised, but a negative exercise test does not exclude a diagnosis if other sentinels such as syncope, family history or positive genetics are present. Importantly, exercise is a trigger for malignant arrhythmias, and caution is advised; however, beta-blocker therapy, the avoidance of certain medications, adequate hydration, and receiving expert monitoring and treatment can enable some patients to participate in competitive sports.^29,86,89^

### Congenital heart disease and arrhythmias

Arrhythmias are a common comorbidity in CHD, both before and after surgical procedures. In patients with CHD, arrhythmia assessment should be an integral part of routine evaluation, which should always include assessment during exercise.^9^ Exercise prescription and advice require individualised, patient-specific risk stratification.^9,90–92^

A major challenge in screening is the presence of undiagnosed structural congenital heart conditions that might predispose individuals to arrhythmias such as anomalous origin of a coronary artery, which can result in life-threatening ventricular arrhythmias during strenuous exercise.^93^ Advice for competitive sports eligibility and assessment for patient athletes with CHD should be followed.^9,10^

### Acquired arrhythmias in paediatric athletes

Acquired arrhythmias, though less common in paediatric athletes, are a significant risk for sudden cardiac events. These arrhythmias can manifest as bradyarrhythmias, including sinus node dysfunction and various degrees of AV block, or as tachyarrhythmias, either supraventricular or ventricular in origin.

Myocarditis, often caused by viral infections, is a common cause of acquired arrhythmias. Myocarditis can present with ventricular arrhythmias (leading to palpitations or syncope), and/or AV conduction abnormalities, including complete AV block. Additionally, myocarditis can trigger early-onset atrial fibrillation, even though this condition is typically associated with long-term high-intensity endurance training and is rare in young athletes without underlying abnormalities.^94^ Myocarditis is a cause of myocardial fibrosis in children, a risk factor for exercise-induced arrhythmias.^95^

Myocarditis is an under-reported cause of sudden cardiac death.^96^ During the acute phase, athletic individuals with a probable or definitive diagnosis of recent myocarditis should abstain from competitive sports or leisure sports while active inflammation is present.^10^ A structured return-to-play after 3–6 months is possible, after a comprehensive clinical evaluation, and with a case-by-case decision. In these cases, athletes should be asymptomatic, with normal troponin and biomarkers of inflammation, normal LV systolic function, no evidence of ongoing inflammation or myocardial fibrosis on cardiac magnetic resonance (CMR) imaging, good functional capacity, and absence of frequent and/or complex ventricular arrhythmias.^10,97^

Rheumatic heart disease and Chagas disease can result in arrhythmias in paediatric athletes, particularly in developing countries. Other less common causes include Kawasaki disease, cardiac tumours, and cardiac involvement in systemic diseases such as lupus, sarcoidosis or systemic sclerosis.^29^

Evaluating arrhythmias related to acquired heart disease requires comprehensive ECG monitoring, advanced imaging, and, when necessary, electrophysiological studies. Timely identification and management are critical to ensure the safety of paediatric athletes in sports.^29^

## Echocardiographic evaluation: appropriateness criteria, indication, protocol, reference values, scaling

Transthoracic echocardiography is an important imaging modality used to identify structural heart diseases associated with an increased risk of SCA/D, including cardiomyopathies, coronary artery anomalies and aortic and valvular disease.^98^ As shown in *Table 2*, an echocardiogram is indicated in the presence of: (1) abnormal ECG findings; (2) abnormal physical examination (murmurs or stigmata of connective tissue disorders); (3) family history of cardiomyopathies or SCA/D < 40 years old; or (4) cardiovascular symptoms during exercise.^99^

A single echocardiographic screening in competitive athletes during adolescence to exclude the presence of mitral valve prolapse, bicuspid aortic valve, aortic enlargement and coronary anomalies is proposed in some papers.^100^ This document suggests this approach may be appropriate, provided expertise and resources are available. The cost-effectiveness of this strategy is still debated.^101^ At present, there is no evidence-based answer to the question of the frequency of echocardiographic follow-up in asymptomatic paediatric athletes. The clinical manifestation of cardiovascular disease is variable and may not be diagnosed by a single echocardiographic evaluation.^98^

For correct echocardiographic quantification of cardiac structures, it is important to refer to age, gender, genetic background and body size-specific nomograms (z-scores).^102–104^ In addition to standard measurements, there has been a growing interest in the use of myocardial deformation imaging (speckle-tracking echocardiography) and 3D echocardiography to differentiate between the physiological adaptation to exercise and the early stages of cardiomyopathies.^99,105,106^

Exercise stress echocardiography can be used to investigate symptoms during exercise or to differentiate between cardiomyopathies and exercise-induced myocardial remodelling in individuals with a minimum height of 140 cm.^107^ It allows contractile reserve assessment, specifically in paediatric athletes^108^ in the grey zone where it may be hard to discern between athletic remodelling and early cardiomyopathy. Additionally, it can assess valvar stenosis or regurgitation and its response to exercise and assess RV and pulmonary pressures. Exercise echocardiography, or where available, exercise or pharmacological stress CMR should be performed to detect subclinical signs of myocardial ischaemia.^86,109–111^

Echocardiography can identify structural CHD in the majority of cases. However, computed tomography (CT) provides better morphological visualisation of the entire coronary artery system and might be used in selected cases, as coronary artery abnormalities are only detectable in 85–90% of cases by focused echocardiography with high-resolution probes, dedicated settings and specific expertise.^110,112,113^ If echocardiography is inconclusive and suspicion exists, CMR imaging is advised due to its ability to provide non-invasive, high-resolution images of coronary artery anomalies without radiation exposure.^114^

## Echocardiographic evaluation: LV exercise-induced LV remodelling and reference values of LV size, thickness (including z-scores) and function

Exercise-induced changes of the left ventricle are less evident in paediatric athletes compared to adults, but are still significant when compared with paediatric sedentary subjects.^1,115^ Adaptations occur even in pre-pubertal athletes following a relatively short period of training.^116^ Specifically, a 10-week endurance training programme was sufficient to determine exercise-induced LV remodelling with a significant increase in wall thickness, LV end-diastolic diameter and LV mass in naïve male adolescents.^117^ In pre-adolescent athletes (12 years old), the initial LV remodelling is characterised by an increase in the LV wall thickness, LV mass and relative wall thickness, determining a concentric LV remodelling without a marked increase in LV volumes.^118^ In a prospective 3-year follow-up study,^119^ continuing competitive endurance sport induced a subsequent increase in the LV end-diastolic diameter, without further significant changes in wall thickness and LV mass, resulting initially in a normalisation of LV geometry and later in eccentric remodelling.^120^

In early adolescence, a potential grey zone ranging from a septum thickness z-score of 2.0 to 3.3 can be identified in a similar proportion of paediatric athletes and HCM genotype-positive patients,^120,121^ thus impairing the differential diagnosis. Therefore, longitudinal echocardiographic follow-up is mandatory in those cases falling in the grey zone, as a 3-year prospective study demonstrated that LV hypertrophy progresses only in HCM genotype-positive patients, while athletes developed larger LV volumes throughout the study period without worsening hypertrophy.^121^

It is advised to use body surface area (BSA)-adjusted z-scores of over 2 standard deviations above the mean/median value for age instead of the absolute value, as this accounts for the rapid growth spurt in puberty. Specific z-score nomograms for LV dimensions, wall thickness and mass in paediatric athletes at present are limited to Caucasian^104,122^ and Arab/Black^102^ male soccer athletes.^123,124^

Systolic and diastolic LV function are normal in paediatric athletes, both at rest and at maximal effort. A superior stroke volume index, cardiac output and E’ wave velocity have been described at maximal effort in pre-adolescent soccer players mediated by a reduction in the heart rate coupled with a greater allometrically scaled LV end-diastolic volume.^61,116^

Sex and ethnic differences are present during adolescence. Female athletes show less pronounced exercise-induced LV remodelling^125^ while Black athletes show the greatest LV hypertrophy, compared to White and mixed-race athletes. Nevertheless, only 7.1% of Black athletes have a LV wall thickness >12 mm compared to 5.9% of mixed-race and 1.3% of White athletes.^126^

## Echocardiographic evaluation: RV exercise-induced RV remodelling and reference values of RV size (including z-scores) and function

Exercise-induced remodelling of the right ventricle in athletes encompasses both structural and functional adaptations, influenced by variables such as sport type, training intensity, age, sex, and ethnicity.^1^ To accurately evaluate RV size, function, and wall thickness, multiple standardised echocardiographic views, including parasternal, RV-focused apical 4-chamber, and subcostal views, should be employed, following established echocardiographic protocols.^103^ As in the left ventricle, a critical aspect of echocardiographic assessment is differentiating between physiologic RV enlargement associated with athletic training and pathologic enlargement due to underlying structural heart disease. Disproportionate RV dilatation relative to the left ventricle, reduced global RV systolic function, and the presence of regional wall motion abnormalities may indicate underlying RV pathology.

Paediatric athletes generally exhibit larger cardiac dimensions compared to non-athletes, even after adjusting for age.^1,118,127^ RV remodelling is evident across a range of sports, with larger dimensions typically observed in endurance athletes compared to non-endurance athletes. As for the left ventricle, z-score nomograms accounting for age and BSA should be used.^1,103^ However, specific nomograms for the right ventricle are not currently available for paediatric athletes. Therefore, when evaluating the right ventricle in paediatric athletes, the following factors should be taken into account: BSA, age, sporting discipline, level of athletic conditioning^119^ and the amount of training performed.^120^

In a study involving 76 pre-adolescent cross-country skiers (mean age 12.1 ± 0.2 years), athletes demonstrated significantly greater RV dimensions compared to non-athletes, suggesting that cardiac remodelling begins before puberty.^118^ From puberty onwards, sex differences in RV remodelling become apparent, with males showing more pronounced changes.^125^ By the age of 18 years, 63% of highly trained endurance athletes exceed the upper reference limits for indexed RV end-diastolic area,^120^ with weekly training hours moderately correlating with RV dimension changes (R = 0.47 for RV end-diastolic area and R = 0.53 for RV end-systolic area).^120^ Zaidi *et al*. provide reference values for the upper range of RV measurements in athletes, corrected for sex and BSA, which are applicable to adolescent athletes.^128^

RV systolic function comprises the evaluation of global and regional kinetics. In athletes, global RV function is typically assessed using metrics such as RV fractional area change, tricuspid annular plane systolic excursion and RV global longitudinal strain or free wall strain.^31,32^ RV global longitudinal strain in particular has recently been shown to differentiate between pathology and athletic adaptation in paediatric athletes with a dilated right ventricle.^31^ As in highly-trained adult athletes, both prepubertal and adolescents athletes^31^ may show lower RV systolic function values than non-athletes, a finding that mirrors observations in adult athletes and is likely attributable to the increased haemodynamic load on the right ventricle during exercise.^129^ However, values below normal could raise the suspicion of an underlying cardiomyopathy.^31^ In the case of marked RV remodelling (dilatation and slightly reduced RV systolic function), exercise imaging could be helpful for differentiating physiology (preserved RV contractile reserve) from pathology (blunted or decreased RV increase with exercise), although data in paediatric athletes are still scarce.^130,131^

## Echocardiographic evaluation: atria and aorta. Biatrial remodelling, aortic dimensions, reference values and z-score

Biatrial remodelling is a recognised marker of cardiac disease associated with an increased cardiovascular risk.^132^ However, training-induced biatrial dilatation is a physiological adaptation of the heart in response to the stimulus of exercise and is accompanied by a normal atrial function, different from pathological remodelling.^133^ While biatrial remodelling has been extensively investigated in adult athletes, data on paediatric athletes are scarce. However, it has been demonstrated that training—and particularly endurance training—can induce biatrial enlargement in pre-adolescents, suggesting that the growing atria in children can also be remodelled by exercise.^104,134–136^ Notably, this remodelling was associated with normal atrial strain values and normal values of biatrial ejection fraction and correlates with stroke volume, supporting the interpretation of this phenomenon as a physiological exercise-induced adaptation of the heart.^118,134^

Given that mild biatrial dilatation is normal in pre-adolescents, it is crucial to interpret echocardiographic data in this population appropriately. However, further data are needed to establish normative reference values of biatrial size, volume and function, taking into account the type of sport (with endurance sports leading to the most significant degree of remodelling), years of training, training volume per week, BSA and sex.

In adult elite athletes, the absolute and index dimensions of the aortic root are slightly enlarged compared to non-athletes, but still fall within the established limits for the general population (>40 mm for male athletes, >34 mm for female athletes). Age, sex, ethnicity and sporting discipline are also relevant, with the highest impact from sports with a high dynamic component in both sexes.^137^ This was also confirmed in athletes with a bicuspid aortic valve compared to non-athletes.^138^

Very limited data exist in paediatric athletes, but a recent meta-analysis^1^ confirmed a +10.4% increase in aortic dimensions in junior athletes vs non-athletes, which increases with age (+14.2% when comparing ≥14 vs <14 years) and in Black junior athletes (+9.4%) vs Caucasian. Dimensions of the aortic root, ascending aorta and sino-tubular junction should be expressed in absolute values and z-scores (instead of values indexed to BSA), to more precisely follow up over time. Specific nomograms for atrial and aortic root dimension have been published only in Caucasian and Black/Arab male soccer athletes,^102,104^ while female athletes and other sporting disciplines are still underrepresented in research data. Assessment of the coronary artery origin should be part of a screening echocardiogram, and if there is suspicion of abnormality, cross-sectional imaging should be performed.

## The role of exercise testing and mobile ambulatory ECG monitoring

### Exercise testing

The exercise stress ECG is an important test for paediatric athletes, both for diagnostic and prognostic evaluation. It is advised in the following situations:^139^

1. in the presence of exercise-induced symptoms (including palpitations, syncope or near-syncope, dyspnoea, undefined malaise associated with pallor during or immediately after exercise, etc.);
2. in the case of an abnormal ECG at rest, which raises suspicions of a concealed form of cardiomyopathy or a channelopathy;
3. to rule out the presence of exercise-induced arrhythmias;
4. in the presence of a known cardiovascular condition, to rule out an abnormal response to exercise and assess disease progression;
5. to prescribe a personalised exercise training programme; or
6. to evaluate the return to competitive sport after a specific medical or surgical treatment for a cardiovascular condition.

The cardiopulmonary exercise test (CPET) couples a stress ECG with the analysis of exhaled gases and ventilatory parameters. CPET is the preferred test for the assessment of cardiorespiratory fitness, exercise prescription and in the diagnosis/follow-up of exercise-induced asthma, but also aids in the diagnosis of suspected conditions with decreased heart function, such as cardiomyopathies or structural CHD.^140,141^

Cycle ergometers and treadmills should be height-adjustable, and the appropriate protocol should be chosen based on the walking pace or the pedalling cadence of the child. Nevertheless, performing a stress ECG/CPET in young children is more challenging than in adolescents, not only due to their smaller body size, but also due to possible reduced attention and cooperation, so stress ECG/CPET should be performed by a team with paediatric expertise. A 3-min continuous recording ECG during a Harvard Step Test with an abrupt interruption followed by 3 min of post-exercise monitoring could be a valuable, cheaper and easily available alternative in paediatric athletes to rule out the presence of exercise-induced arrhythmias, as shown in an Italian cohort, where a diagnosis of a cardiovascular pathology at risk of SCA/D was made in 41 (59%) on resting 12-lead ECG, and 36 (52%) on exercise testing.^6^ A short step exercise test has also been used as a primary screening tool and has shown increased diagnostic yield, but also a significant false positive rate.^142,143^ At present, there is insufficient evidence to include it as a primary screening tool.^6^ Exercise stress echocardiography can complement the exercise assessment.

### Mobile ambulatory ECG monitoring

While not useful as a primary screening tool, mobile ambulatory heart rhythm monitoring should be part of any secondary investigation in paediatric athletes with cardiac symptoms or signs of potential disease. Inherited cardiomyopathies are a common underlying cause of SCA/D; however, the mode of presentation is more often an arrhythmia rather than cardiac dysfunction.^144^ Mobile cardiac rhythm monitoring is also extremely helpful in risk stratification and eligibility decision-making in the paediatric athlete with cardiac disease. Newer cordless rhythm monitoring devices are being approved for use in children, and initial data show comparability in accuracy.^145,146^ Ambulatory monitoring should include on-field training and exercise sessions. In some circumstances, a 12-lead ambulatory ECG may be helpful to establish the origin of ectopic or arrhythmic activity.

## Further diagnostic pathways in abnormal findings (cardiac magnetic resonance, coronary computed tomography, familial screening, genetic testing)

Echocardiography remains the primary imaging tool for cardiovascular assessment in the paediatric population; however, other techniques, such as CMR and CT, have become increasingly important when initial evaluations indicate potential cardiac abnormalities associated with SCA/D.

### Cardiac magnetic resonance

CMR is a standard modality in imaging CHD in children, given its capability to simultaneously assess dimension, morphology and function of ventricles, blood flow of valve and great vessels and tissue characterisation without the need for radiation and iodinated contrast agent.^147^ CMR in paediatric CHD patients poses some challenges and nearly all sequences need to be adapted to the patient’s size, age, heart rate and the specific clinical question. Therefore, CMR studies of children should be performed in a centre with specific expertise and adequate equipment.^1,2^ CMR is also indicated for a detailed assessment of morphology, function, and tissue characteristics, helping to distinguish between physiological adaptations and cardiomyopathies.^147^ In addition, CMR is valuable in diagnosing myocarditis, which may present with exercise intolerance, palpitations and atypical chest pain.^147^ Exercise-related cardiac remodelling, as described, also generates additional challenges for cross-sectional imaging interpretation due to the lack of normative reference ranges in paediatric athletes.

The assessment of coronary artery anomalies should be part of the standard CMR evaluation. In paediatric patients, coronary evaluation by CMR is indicated in cases of suspected congenital anomalies of origin, Kawasaki disease, or other acquired coronary pathology.^1,2^ High heart rate and small size remain limiting factors in young children, and in addition, specific anatomic features that are likely to be linked to prognosis, such as ostial narrowing and the intramural course, are better characterised by CT. Exercise CMR remains predominantly a research tool in the paediatric population, but may help in the unmasking of cardiac exercise pathology.^148^

### Coronary computed tomography

Coronary CT in patients of all ages has superior accuracy for coronary artery anomalies because of the ability to simultaneously visualise the coronary arteries as well as the great vessels. While CMR is preferred due to the absence of radiation exposure, especially in younger patients, coronary CT can be invaluable when coronary anatomy is a concern and cannot be visualised by echocardiogram or CMR.^3,4^ Cardiac CT also plays a key role in imaging unrepaired and repaired CHD, with the choice between CMR and CT guided by factors such as the patient’s age, diagnosis, clinical condition, specific clinical questions, and patient preference.

### Genetic testing and family screening

All paediatric athletes with a strong suspicion of an inherited cardiac condition should be referred to an inherited cardiac conditions service for advice on family screening and genetic testing. Genetic testing is advised for phenotype-positive children, while cascade screening within families with a recognised pathogenic variant can enhance risk stratification, allowing introduction of effective preventive therapies and appropriate sports restrictions in specific conditions that carry a high risk of sudden death in childhood and adolescence. Conversely, for phenotype-negative children with a familial diagnosis of cardiomyopathy, the timing of genetic testing should be individualised based on the test's impact on management, consent, and the long-term implications of a positive result. In families with a history of SCA/D, molecular autopsy aids in assessing risk among surviving relatives. According to a document led by the EAPC, recommendations on genetic testing for adult athletes also give relevant guidance for paediatric athletes.^33^

If genetic testing is advised, a referral should be made to a clinical geneticist to ensure expert counselling and follow-up.^149^ Genetic testing in minors involves highly specialised medical and ethical issues, and all paediatric athletes with a strong suspicion of an inherited cardiac condition should be referred to an inherited cardiac conditions service for advice on family screening and genetic testing.^149^

## Legal responsibilities: the complex relationship between physician, parents, paediatric athletes and coaches

There is a complex relationship between paediatric athletes, parents, physicians and coaches. In the context of cardiac screening, legal issues may arise in relation to consent, confidentiality and liability for major adverse events.

Consent must be obtained for screening, either from the athlete and/or a parent.^150,151^ Specific rules vary by jurisdiction and may depend on the athlete’s age and/or maturity. Informed consent involves an understanding of the risks and benefits of the test, the accuracy and the potential implications of an abnormal finding.^49,152^ In the UK, children under 16 years can consent if they are sufficiently able to understand what is involved (known as *Gillick* competence).^153,154^ Some European countries also set a threshold based on maturity, while others specify a minimum age.^155^ For more major medical decisions (e.g. surgery or return to play with a cardiovascular condition), parental consent should be obtained.

Explicit consent from the athlete or parent is required to share medical/screening information with the club or coach.^156^ Difficulties can arise if an athlete does not wish to share some or all details of a cardiac diagnosis with a club, and this situation must be handled with extreme care. There must be a clear understanding of who is responsible for communicating the results of screening (including subsequent investigations) to the athlete. While physicians may face conflicting pressures/demands from parents and coaches, the doctor’s primary duty is always to the patient.^150^

Liability issues may be complex if an athlete suffers an adverse medical event. Legal action often involves a number of defendants (club, physicians and others) and liability may be apportioned between multiple parties. Consequently, physicians may be more restrictive in their advice. This consensus document aims to provide evidence-based guidance to support physicians advising young athletes.

Finally, the ethical implications of screening in the paediatric population are important and distinct from those of adult athletes. One benefit of screening in the paediatric age group is that pathology may be identified before an individual is on an elite, semi-professional track and may be redirected to another sport or profession earlier. However, the lack of autonomy, patient consent issues and potential lifelong commitment to surveillance are key factors which must be taken into account when implementing cardiac screening in paediatric individuals. It is important that physicians involved in evaluation, diagnosis and management have expertise specific to paediatric athletes to ensure advice is appropriate, accurate and timely. All decision-making should follow a multidisciplinary team approach, with the paediatric athlete at the centre.

## Sport participation while awaiting further evaluation

Abnormal findings in cardiac screening do not always preclude continued participation in competitive sport. A decision should be made on an individual case-by-case basis by the screening physician, and if required, after discussion with an expert (paediatric) cardiologist in a multidisciplinary team approach to assess the risk of SCA/D during sport while an athlete is being investigated.

## Conclusions, advice and future directions

Until now, advice for cardiac screening in paediatric athletes was primarily guided by guidelines designed for adult athletes. These guidelines relied on data derived from studies involving adult athletes^2^ with suboptimal accuracy. This document introduces specific advice for paediatric athletes for the first time, based on expert consensus, and where applicable, data from paediatric athlete populations.

The proposed ECG thresholds (normal, borderline, abnormal) are an important step forward in advancing knowledge of specific ECG criteria for paediatric athletes. These thresholds require prospective validation in paediatric athlete cohorts, with both sexes and different ethnicities represented. In addition, the diagnostic accuracy of the proposed paediatric ECG thresholds should be assessed to validate their use in paediatric athletes.

The necessity for such a document arises from the specifics of cardiac physiology, maturation and growth, age-related disease expression, modified diagnostic pathways, and training adaptations. Medico-legal aspects for paediatric athletes also differ from those of adult athletes and are not adequately addressed in available adult-focused documents.

At the same time, current adult cardiac screening guidelines have had a significant influence on this document, and the aim was to incorporate those to create a consistent approach in cardiac screening throughout childhood to adulthood. It is apparent that progress has been made in the cardiac care of paediatric athletes in recent years, but further research is required to optimise screening strategies, accurately assess and quantify the risk of SCA/D and provide evidence-based eligibility recommendations for paediatric athletes with cardiac disease.

Finally, the education and training of health care professionals to obtain paediatric sports cardiology expertise are currently informal, and too few opportunities are available. Academic and sports organisations, policymakers, and paediatric medical and sports professionals will all need to work together to provide appropriate medical care for the paediatric athlete population.

## Declarations

### Data Availability

No data were generated or analysed for or in support of this paper.
